# Updates in Diagnostic Techniques and Experimental Therapies for Diffuse Intrinsic Pontine Glioma

**DOI:** 10.3390/cancers17060931

**Published:** 2025-03-10

**Authors:** Luke McVeigh, Tirth Patel, Madeline Miclea, Kallen Schwark, Diala Ajaero, Fareen Momen, Madison Clausen, Tiffany Adam, Rayan Aittaleb, Jack Wadden, Benison Lau, Andrea T. Franson, Carl Koschmann, Neena I. Marupudi

**Affiliations:** 1 Department of Neurosurgery, Michigan Medicine, Ann Arbor, MI 48109, USA; lmcveigh@med.umich.edu; 2 Department of Pediatrics, Michigan Medicine, Ann Arbor, MI 48109, USA; tirth@med.umich.edu (T.P.); micleam@med.umich.edu (M.M.); kmschwar@med.umich.edu (K.S.); dialala@med.umich.edu (D.A.); fmomen@med.umich.edu (F.M.); clausema@med.umich.edu (M.C.); tiffadam@umich.edu (T.A.); rayanait@med.umich.edu (R.A.); wadden@umich.edu (J.W.); lbenison@med.umich.edu (B.L.); ckoschma@med.umich.edu (C.K.)

**Keywords:** diffuse intrinsic pontine glioma, DIPG, diffuse midline glioma, DMG, brainstem biopsy

## Abstract

Diffuse intrinsic pontine glioma (DIPG) is a rare but extremely malignant central nervous system tumor primarily affecting children that is almost universally fatal with a devastating prognosis of 8-to-12-month median survival time following diagnosis. Traditionally, DIPG has been diagnosed via MR imaging alone and treated with palliative radiation therapy. While performing surgical biopsies for these patients has been controversial, in recent years, advancements have been made in the safety and efficacy of surgical biopsy techniques, utilizing stereotactic, robotics, and intraoperative cranial nerve monitoring as well as the development of liquid biopsies that identify tumor markers in either cerebrospinal fluid or serum. With more molecular data being collected from these tumors due to more frequent biopsies being performed, multiple treatment modalities including chemotherapy, radiation therapy, immunotherapy, and epigenetic modifying agents continue to be developed. Numerous recent clinical trials have been completed or are currently ongoing that have shown promise in extending survival for patients with DIPG. Focused ultrasound (FUS) has also emerged as an additional promising adjunct invention used to increase the effectiveness of therapeutic agents. In this review, we discuss the current evidence to date for these advancements in the diagnosis and treatment of DIPG.

## Simple Summary

Diffuse intrinsic pontine glioma is an infiltrative and aggressive tumor, primarily affecting children, and is resistant to standard therapies, contributing to its poor and fatal prognosis. Current research in treatments for diffuse intrinsic pontine gliomas are focused on understanding the genetic mutations and molecular biology of the tumor in order to develop novel targeted therapies. Advanced treatment modalities include immunotherapy, targeted drug treatments, and epigenetic modifying agents. Advances in stereotactic brainstem biopsy techniques and exploration of drug delivery methods that disrupt the blood–brain barrier (BBB) continue to advance treatment methods towards more effective and personalized treatment options, offering hope for improving survival rates and quality of life for affected children.

## 1. Introduction

Diffuse intrinsic pontine glioma (DIPG) is a devastating central nervous system (CNS) malignancy primarily affecting pediatric patients. DIPG is the second most common malignant CNS tumor in children affecting roughly 150–400 children per year in the United States with a peak incidence at ages 6–9 years old [1,2,3,4]. Despite research efforts over the past few decades, the diagnosis carries an abysmal prognosis with a median survival of just 8–12 months and a 2-year survival rate of 10% [5]. The current standard of care for treatment is radiation therapy; however, radiation serves only as palliative care and is not curative. DIPG is an invasive, malignant neoplasm that is classically found in the pons arising from astrocytes, though also contain oligodendrocyte precursor cells that are highly infiltrative and proliferative [6]. These tumors were initially discovered in the brainstem; however, similar tumors have been found in the cerebellum as well as the thalamus and other midline structures leading to a reclassification of the tumors as diffuse midline gliomas (DMGs) [7,8]. The most recent World Health Organization (WHO) CNS tumor classifications (2021) include four general groups of diffuse gliomas: adult-type diffuse gliomas, pediatric diffuse low-grade gliomas, pediatric-type diffuse high-grade glioma, and circumscribed astrocytic gliomas. Molecular features primarily define pediatric-type diffuse high-grade glioma which include diffuse midline glioma, H3K27 altered; diffuse hemispheric glioma, H3 G34-mutant, H3-wildtype, and IDH-wildtype; and infantile-type hemispheric glioma. Tumors historically known as DIPG have been reclassified as a subset of DMGs now termed “diffuse midline glioma, H3 K27M-altered” and fall in the category of pediatric diffuse high-grade glioma [9,10].

Diagnosis is typically made by a clinical presentation with symptoms such as cerebellar dysfunction, myelopathy, or cranial nerve palsies in combination with imaging findings on MRI of the brain classically including a mass located in the pons that is T1 hypointense, T2 hyperintense with or without some amount of contrast enhancement, sometimes peripherally enhancing with central areas of necrosis and/or edema [11]. Due to the precarious location and lack of treatment options, surgical resection and even biopsies of these lesions have not been common practice, thus leading to a lack of molecular data for these tumors. In recent years, with innovation of stereotactic and robotic biopsy techniques as well as advancements in precision medicine focused on specific molecular markers, the push for biopsy to be a part of the work up for these patients has increased. In this review, we discuss the latest advancements including the role and safety of stereotactic biopsy for these patients and the experimental therapies that are currently undergoing investigation for the treatment of this difficult disease (Table 1).

## 2. Surgical Intervention/Biopsy

Historically, DIPG was a radiographic diagnosis made via MR imaging with distinct characteristics. Many in the field of pediatric oncology and neurosurgery have argued against the use of biopsy in the diagnosis of DIPG given the high-risk location and minimal to no benefit for the patient. While controversial, stereotactic biopsies of the brainstem have been in use for decades, first reported in 1978 by Gleason et al. [12]. Despite the early development of more precise and minimally invasive biopsies with stereotactic techniques, the sampling of tissue for patients with DIPG was hotly debated with Albright et al., in 1993, arguing against biopsy as standard of care given the results did not change clinical management, and diagnosis could still be made reliably with MRI alone [13]. Since these early papers on the topic, the advancement in the understanding of the molecular biology to characterize DIPG lead to identifying the H3K27M mutation in the histone H3 gene. The identification of this key mutation provided additional rational for tumor biopsy as it provided a potential therapeutic target and assisted with prognostication. Prior to identifying the H3K27M mutation, the reasoning for biopsy included diagnostic confirmation, confirmation in cases of unusual imaging appearance, or the altruistic behavior of the family/caregiver in allowing tissue donation for research purposes. While there remains controversy regarding biopsies, due to the advancing knowledge on the molecular characterization of DIPG, performing biopsies for children with DIPG has become more standard for many groups, allowing for enrollment in one or more clinical trials that have shown promise for improving life expectancy by weeks to months. There have now been a number of clinical trials investigating the safety and diagnostic yield of these biopsies.

The surgical technique for performing a tissue biopsy typically entails creating a small burr hole or twist drill hole. The entry site for the burr hole and target site for the tissue biopsy are determined based on a stereotactic or navigation-assisted system. A stereotactic needle is inserted through the burr hole along the planned trajectory to the target. The stereotactic needle allows for small core biopsies to be obtained via a gentle aspiration technique. There are two widely used trajectories for biopsies of the brainstem, the infratentorial transcerebellar approach and the supratentorial transfrontal approach [14]. Of these two approaches, the transcerebellar option is most commonly used to biopsy DIPG. The transcerebellar approach is often preferable for DIPG as it has a shorter trajectory with the biopsy needle entering through the posterior fossa and transversing the cerebellum into the middle cerebellar peduncle to reach the pons. This approach is not only shorter but also transverses less eloquent structures than those of the transfrontal approach [15,16]. The primary limitation of this approach is it can only access lesions in the pons and upper medulla; therefore, it may not be suitable for other DMGs that extend to other locations. The supratentorial transfrontal approach has the advantage of allowing access to more areas of the brainstem including the midbrain; however, this approach requires a much longer trajectory to target. The trajectory includes transversing the frontal lobe, corpus callosum, and deep brain structures to reach the brainstem. These trajectories must be carefully planned to avoid important vasculature, the ventricular system, and the tentorium. With both approaches, trajectory planning software is a vital tool in establishing a safe path to obtaining a pathology sample of diagnostic value.

In recent years, the frequency of biopsies for DIPG have increased as more groups have found it valuable to obtain pathological information for multiple reasons. There are cases with irregular characteristics on the imaging that require clarification of diagnosis with a tissue sample. Additionally, tissue sampling can provide further information into why children diagnosed with DIPG have some variance in length of survival, as well as immunohistochemical identification of biomarkers that may be targets for current and future therapies. With more robust data displaying the safety of brainstem biopsies in children in combination with more experimental therapeutic targets being identified, the field is becoming more accustomed to obtaining a biopsy of these lesions as a part of the standard work-up.

While stereotactic biopsies of the brainstem were initially introduced in 1978, not many studies reporting the safety and efficacy of biopsies were published in the following decades until the turn of the century, likely due to the low number of biopsies performed [12]. The earliest studies reporting on the safety and diagnostic utility of brain biopsies in children were predominantly studies with a mixed population of adults and children with brain stem lesions and were not exclusive to DIPG [17]. Over the past two decades, there has been a significant increase in the number of studies reporting data on biopsies conducted for DIPG in children. In 2023, Dalmage et al. performed a meta-analysis reporting the morbidity and mortality of biopsies for DIPG as well as the diagnostic yield of stereotactic biopsies [18]. This meta-analysis identified 14 studies, primarily case series and cohort studies totaling 192 cases, specifically investigating the safety and efficacy of biopsies for children with DIPG, including a combination of studies utilizing frame-based, frameless, and robot-assisted stereotactic biopsy techniques via both transfrontal and transcerebellar approaches [19,20,21,22,23,24,25,26,27,28,29,30,31]. This study reported a combined complication rate of 13% (25/192 patients) with complications including cranial nerve palsy, intracranial hemorrhage, hemiparesis, dysarthria, dysphagia, and new or worsening ataxia. Cranial nerve palsies were the most frequently reported complication, with cranial nerves VII and VI most commonly affected. There were no cases of cerebrospinal fluid (CSF) leak, infection, or death specifically attributed to the biopsy reported in the literature. A year prior, in 2022, Lu et al. performed a meta-analysis investigating the safety and efficacy specifically for frameless robotic-assisted biopsy of pediatric brainstem lesions [32]. Their study identified 8 cohort studies totaling 99 patients and found a pooled estimated complication rate of 10% [20,21,22,31,33,34,35,36]. Of note, all complications in these studies were transient, and the rate of permanent complication of biopsy was 0%. The efficacy of achieving diagnosis was reported at 100% for these cases. Since the publication of these meta-analyses, one additional retrospective cohort study by Lim et al. reported their experience with the safety and efficacy of biopsy of intrinsic brainstem tumors [37]. Of their 11 patients undergoing stereotactic biopsy, no post-operative complications were reported. Based on these data reported in the literature, performing a biopsy for suspected DIPG appears to be a relatively safe procedure with particularly low risk of permanent deficit or mortality, especially with new and developing techniques such as robot-assisted biopsies.

In addition to advances in biopsy trajectory planning with computer software and assistance from robots to perform biopsy, other advancements such as intraoperative monitoring are continuing to be developed to make biopsies of the brainstem safer. A few neurosurgical groups have begun to utilize intra-operative stimulation that is integrated into the biopsy probe. This monitoring allows for the surgeon to recognize when encountering eloquent tissue and adjust the trajectory if needed to avoid causing transient or permanent deficits. There are a limited number of studies investigating the safety and efficacy of this technique, but early results are promising. Labuschagne et al. reported a small case series utilizing an intraoperative stimulating probe integrated into a standard stereotactic biopsy needle in nine pediatric patients [25]. Of these nine cases, two of them changed their trajectory based on the intra-operative monitoring feedback, and none of the nine cases experienced any post-operative complications. The further development of this technique may lead to even safer biopsies allowing for wider adoption of biopsy as a standard of care for patients with suspected DIPG.

## 3. Liquid Biopsy

While morbidity rates from stereotactic biopsy of pontine high-grade gliomas are low when performed at experienced surgical centers, universal access to DIPG-specific care is limited, and biopsies are still generally considered high-risk [38]. Additionally, tracking the progress of pontine diffuse midline gliomas via radiographic imaging can be problematic due to their diffuse nature and radiation-induced swelling (e.g., pseudoprogression [39]). In light of these challenges, liquid biopsies have emerged as a clinically relevant, minimally invasive tool to aid in the diagnosis and monitoring of DIPG [40]. Liquid biopsy refers to the analysis of tumor-derived biomarkers present in disease-relevant biofluids such as CSF and blood. Tumors are known to release tumor-specific biomarkers either actively (e.g., circulating tumor cells or extracellular vesicles) or passively upon death via apoptosis or necrosis (e.g., DNA and RNA). These biomarkers enter into the interstitial fluid of surrounding tissue and then propagate to the CSF, blood, and urine where they are decomposed or filtered for excretion. Liquid biopsy of CNS tumors is unique from other diseases in that the blood–brain barrier (BBB) selectively filters tumor-related biomarkers, generally increasing their relative concentration in CSF and decreasing their concentration in the blood.

High levels of tumor-related biomarkers have been found in CSF in a variety of studies [40,41,42,43,44,45]. Because up to 80% of DIPGs are characterized by the H3K27M mutation, ultra-sensitive droplet digital PCR (ddPCR) assays have been developed that can both detect and track cell-free circulating DNA (cf-tDNA) levels [46]. CSF c-ftDNA levels range from undetectable to very high (>10% variant allele fraction) and vary by the location of sample collection [40]. CSF cf-tDNA levels also change depending on treatment stage [40] and the location of sample collection [43,47]. Bruzek et al. applied nanopore sequencing to monitor CSF cf-tDNA levels of multiple primary DIPG tumor mutations and found that CSF cf-tDNA levels tracked with measured tumor area in one case [44]. Cantor et al. found that over a larger set of measurements, absolute tumor size did not correlate with cf-tDNA levels but relative changes in cf-tDNA levels correlated with progression-free survival, with decreases from baseline correlating with extended survival [43]. While CSF is clearly enriched for DIPG biomarkers, pediatric lumbar punctures require general anesthesia and are usually timed to coincide with obtaining magnetic resonance imaging (MRI) of the brain and spine, which also generally require anesthesia. Ommaya reservoirs are small reservoirs placed under the skin outside of the skull that are connected to catheters that give direct access to ventricular CSF [48]. The primary uses for Ommaya reservoirs is relieving intracranial pressure and intracranial administration of chemo- or immunotherapies [45,49], but they also allow for routine, minimally-invasive access to CSF for liquid biopsies [45]. Ommaya placement is well-tolerated and can be performed alongside biopsy [50] but is not part of the current standard of care.

While CSF is well known to be enriched with DIPG biomarkers, blood is much easier to collect, especially in pediatric patients, and is thus a much more desirable biofluid for analysis. However, CSF volume is about 2–5% of that of blood and so even if biomarker leakage was constant and predictable, biomarker levels in the bloodstream are highly diluted. Exactly how biomarkers enter the bloodstream from the CSF is also not well understood and almost certainly varies by tumor size, location, and tumor biology. Regardless, reliable and rapid detection of H3K27M via a blood test would transform clinical trial enrollment. Currently, patients often start radiotherapy before H3K27M diagnoses from stereotactic biopsy (2–3 days to return H3K27M immunohistochemistry) or clinical-grade CSF sequencing results are returned (>2 weeks after lumbar puncture). Rapid ddPCR-based detection of cf-tDNA in plasma has a fairly high sensitivity rate when performed properly [40] and could return clinically relevant diagnoses within 1 day of blood receipt. Additionally, Cantor et al. found that relative changes in the levels of cf-tDNA in blood correlate with progression-free survival (PFS), leaving the door open for future clinical-grade tests to monitor and guide clinical care [43].

cf-tDNA is the most studied liquid biomarker for DIPG-based liquid biopsy due to the large “toolbox” of molecular biology techniques to enrich, amplify, and quantify tumor-specific strands. However, cf-tDNA is in extremely low concentrations relative to other tumor-derived biomarkers. For example, for each tumor cell with a single mutant copy of H3K27M, there are almost certainly >10^6^ of mutant or aberrantly modified histones. Shema et al. developed a technique to profile the epigenetics of plasma-isolated nucleosomes (EPINUC). EPINUC was able to identify nucleosomes containing H3K27me3 histones at a single molecule resolution from <1 mL of plasma [51].

While CSF has a high-concentration of tumor-derived biomarkers, this does not mean that plasma should necessarily be considered “inferior”. Measurable cf-tDNA in the bloodstream could indicate unique biological changes in a tumor like increased migration and/or invasion and provide orthogonal information about BBB compromise and progression that CSF-derived biomarkers would not. Additionally, improvements to the sensitivity of cf-tDNA detection methods (e.g., ultra-ddPCR [52] and rapid error-corrected sequencing [53]) would also expand the utility of blood-based assays. While many questions remain surrounding how liquid biopsy will be used to inform patient care, it is clear that both CSF and plasma have high potential utility. Several barriers remain preventing clinical adoption of liquid biopsies. Mostly, adoption is hindered by a lack of low-cost, rapid, and highly sensitive CLIA-certified assays. Without rapid clinical-grade assays, patients and their families are less likely to request lumbar punctures as any results could not be used to inform patient care. Thus, CLIA certification of established research-grade tests should be of the highest priority to the DIPG community.

## 4. Recent and Ongoing Therapeutic Clinical Trials

Cytotoxic chemotherapy has proven to be largely ineffective in treating patients with H3K27M DMG. The BBB remains a significant challenge; however, even regimens that have improved outcomes in adult glioblastoma, such as radiation with concurrent and cyclic temozolide, have not been efficacious in the treatment of patients with DMG [54].

Targeted therapies have been investigated as a potential substitute for traditional chemotherapy (Table 1). ONC201 (Dordaviprone), an imipridone, has shown progress in treating H3K27M-mutant gliomas. ONC201 is an antagonist of dopamine receptor DRD2/3 and allosteric agonist of the mitochondrial protease caseinolytic mitochondrial matrix peptidase proteolytic subunit (ClpP) and disrupts mitochondrial activity [55,56,57]. In a composite analysis of patients with recurrent H3K27M-mutant DMG, an overall response rate (ORR) of 20% was seen with the use of ONC201 monotherapy [58].

**Figure 1:**
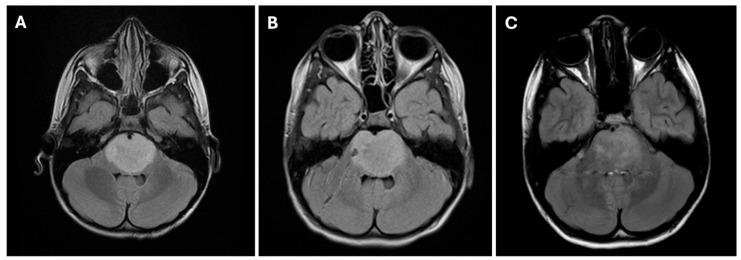
MRI of the brain of a 6-year-old boy diagnosed with DIPG prior to treatment, after biopsy, and after radiation and treatment in targeted chemotherapy clinical trial. (**A**) Axial FLAIR MRI brain demonstrating hyperintense expansile mass consistent with DIPG in the brainstem pons. (**B**) Axial FLAIR MRI brain demonstrating transcerebellar stereotactic needle biopsy track. (**C**) Axial FLAIR MRI brain demonstrating post-treatment reduction in hyperintensity in pons and post-treatment changes after radiation and targeted chemotherapy.

Currently, there is an ongoing phase 2 platform trial utilizing ONC201 combined with paxalisib (PNOC022; NCT05009992). Endpoints in this study center around the efficacy of combination therapy (progression-free survival and overall survival), safety, and toxicity. ONC206, an analogue of ONC201, holds similar molecular characteristics with more potency. The anti-proliferative efficacy of ONC206 has been promising due to its ability to induce cell stress, subsequently inhibiting cellular adhesion and proliferation [59]. The use of ONC206 alone or in combination with radiation therapy is being investigated in a phase 1 study in patients up to 21 years of age with upfront or recurrent DMGs, as well as other recurrent primary malignant CNS tumors (NCT04732065) [60]. Patients receive escalating doses of ONC206 alone or in combination with standard-of-care radiation therapy. Clinical trials utilizing targeted chemotherapy for DIPG aim to improve treatment efficacy while minimizing damage to surrounding healthy brain tissue (Figure 1).

## 5. Targeted Therapies

Increasing use of biopsy and molecular characterization has enabled the use of targeted therapy individualized to additional mutations in the setting of clinical trials. In a randomized biomarker-driven platform trial (BIOMEDE; NCT02233049), newly-diagnosed patients post-radiation from the Innovative Therapies for Children with Cancer (ITCC) clinical trial network, the Brain Tumor Group of the European Society for Pediatric Onocology (SIOPE-BTG), and Australian and New Zealand Children’s Hematology Oncology Group (ANZCHOG) were stratified based on EGFR, mTOR, or no specific biomarker to erlotinib, everolimus, or dasatinib, respectively. A total of 233 patients were enrolled, with a median overall survival (OS) from the date of randomization of 9.0 (95% confidence interval [CI], 7.4–14.4), 11.3 (10.3–13.4), and 9.4 (7.7–10.7) months, for erlotinib, everolimus, and dasatinib, respectively (*p* = 0.45) [61] (NCT02233049). Although the OS was not statistically different from those of historic patient cohorts, this study demonstrated the feasibility of a large, randomized trial for DMG based on molecular characterization from biopsy. Another recent clinical trial (PNOC003, NCT02274987) studied a precision medicine approach in patients aged 3 to 25 years with DMG (specifically DIPG), where patients with newly diagnosed DIPG underwent biopsy and molecular profiling of tumor tissue [62]. A total of 28 patients’ tumors underwent molecular tumor board review, and 19 patients followed treatment recommendations. The median OS was 13.1 (95% CI 11.2–18.4). Importantly, this study concluded that biopsy with rapid molecular profiling was feasible, and molecular results provided insight into potentially targetable alterations and prognostic biomarkers. In an ongoing clinical trial (PNOC008; NCT03739372) using real-time molecular profiling in patients with newly diagnosed high-grade glioma (HGG) in patients ≤ 25 years, 22 evaluable patients enrolled onto Stratum B (non-DIPG DMG), with a median OS of 21.5 months (95% CI 16.8–31.6) [63].

Karyopherin exportin-1 (XPO1), also known as chromosomal region maintenance 1 (CRM1), plays a critical role in carcinogenesis [64]. Selinexor is a new selective oral inhibitor that penetrates the BBB to block XPO1-mediated nuclear export, leading to functional reactivation of tumor suppressor proteins (including TP53, RB1, CDKN1B). An ongoing phase 1/2 trial of selinexor taken in conjunction with radiation therapy in participants with DIPG and HGG patients seek to evaluate the safety, side effects, and recommended phase 2 dose (COG ACNS 1831; NCT05099003) [65].

## 6. Radiation Therapy

Radiotherapy has been considered as the mainstay of treatment for DIPG, with current standard of care treatment for DIPG consisting of external beam radiotherapy with a total dose of 54–60 Gy. Upfront radiotherapy typically results in a temporary improvement in neurologic function and extends OS compared to patients never receiving radiotherapy [66]. Historically, alternative fractionation regimens and/or the addition of radiosensitizers have failed to demonstrate a survival benefit [67]. Currently, a phase 2 trial aims to determine if hypo-fractionated radiation therapy, 25 Gy in 10 fractions, may confer a similar time to progression (SPORT-DMG; NCT05077735).

Optimal reirradiation dose was evaluated in a phase 1/2 trial. Three dose levels (24 Gy in 12 fractions, 26.4 Gy in 12 fractions, and 30.8 Gy in 14 fractions) were evaluated. Clinical improvement was observed, and quality of life was improved in the majority patients with a median OS of 19.5 months and median PFS of 4.5 months from the start of reirradiation [68]. Other retrospective studies published before this study have commonly used a regimen with a 18–20 Gy total dose in 10 fractions and reported a median OS of 3–7 months after reirradiation with an acceptable tolerability [69]. A non-randomized phase 2 trial of the efficacy of conventional fractionation reirradiation with a dose of 30.6 Gy or 36 Gy in recurrent or progressive DIPG is underway (NCT03126266).

Although proton therapy is useful in reducing the radiation dose to healthy brain tissue, studies investigating proton therapy in patients with DIPG are scarce given the poor long-term outcomes in this patient population, which renders patients unlikely to benefit from the advantages of proton therapy.

## 7. Epigenetic-Modifying Therapies

Histone deacetylase (HDAC) inhibitors are compounds that interfere with HDAC enzymes, which play a crucial role in the regulation of gene expression. HDAC inhibitors (HDACi) maintain histones in a hyperacetylated state, keeping chromatin relaxed and transcriptionally active [70]. This hyperacetylated state promotes gene expression, including the reactivation of silenced tumor suppressor genes such as p21^WAF1 and pro-apoptotic genes like BAX and BIM [70,71]. Additionally, HDACi affects non-histone proteins, stabilizing and activating the tumor suppressor protein p53 and modulating critical molecular pathways such as the PI3K/AKT and MAPK pathways to decrease cell survival and proliferation [72,73].

Histone deacetylase inhibitors have been studied preclinically and in clinical trials in patients with DIPG. The HDACi panobinostat was used in H3K27M-mutant glioma models to restore H3K27 methylation and normalize gene expression, thereby decreasing tumor cell proliferation and increasing cell death [74]. Given the preclinical promise, Monje et al. conducted a phase 1 trial, studying panobinostat in patients with DIPG. This trial determined the MTD for two different dosing schedules, with the most common toxicity being myelosuppression [74]. Pharmacokinetic (PK) analysis revealed substantial variability in exposure, suggesting opportunities for further studies to optimize dosing. The PK results highlight the challenges of using targeted therapies in DIPG and underscore the need for exploration of alternative delivery methods and combination therapies to improve treatment outcomes. Of note, further clinical studies with panobinostat were halted with its withdrawal from the US market in 2022.

Dysregulated histone acetylation and vascular endothelial growth factor overexpression have been implicated in pontine high-grade glioma tumorigenesis, suggesting antitumor synergy of HDAC and vascular endothelial growth factor (VEGF) inhibition. The Children’s Oncology Group (COG) ACNS0822 phase 2/3 study evaluated treatment with vorinostat, bevacizumab, or temozolomide monotherapy during radiation therapy, followed by temozolomide and bevacizumab combination therapy. Although adding bevacizumab or vorinostat did not reveal improved clinical outcomes over temozolomide, the study showed improved survival among patients with telomerase-negative tumors, highlighting the potential of targeting telomere maintenance mechanisms as a potential future therapeutic strategy [75].

## 8. Immunotherapy

**Figure 2:**
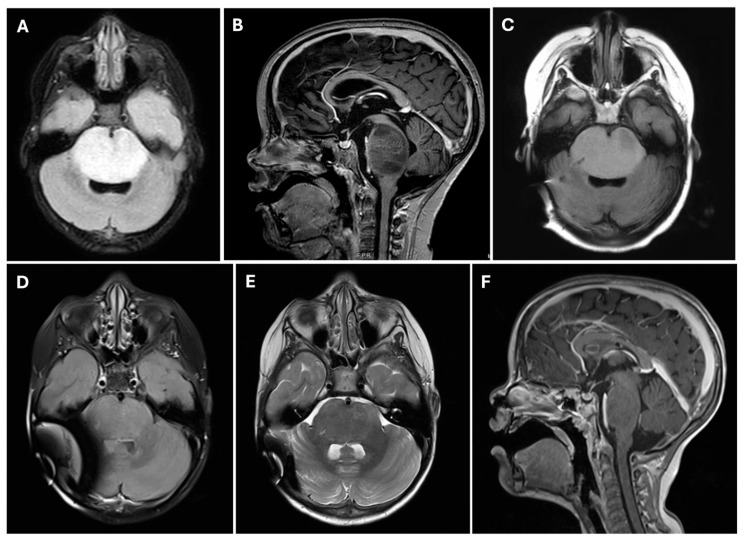
A 4-year-old girl presented with dysconjugate gaze and gain instability. MRI brain images demonstrated an expansile mass of the pons: diffuse intrinsic pontine glioma (DIPG). (**A**) Axial FLAIR MRI of patient with DIPG demonstrating diffuse hyperintensity involving the brainstem pons. (**B**) T1 Sagittal MRI with contrast of a patient with DIPG demonstrating expansile hypointensity of the brainstem pons. (**C**) Stereotactic needle biopsy track along right side of brainstem pons from a transcerebellar approach for DIPG biopsy. (**D**–**F**) Treatment effects of chimeric antigen receptor (CAR) T-cells on diffuse intrinsic pontine glioma (DIPG) at 3 months after the administration of CAR T-cell therapy. (**D**) Axial FLAIR image and (**E**) axial T2 image demonstrating significant reduction in hyperintense signal in the pons and marked reduction in tumor size compared to pretreatment images in Image A. (**F**) Sagittal T1 image with contrast administration demonstrating marked decrease in expansile pontine mass compared to pretreatment images in Image B.

CAR T-cell (chimeric antigen receptor T-cell) therapy utilizes modified T-cell receptor molecules to directly target antigens unique to cancer cells, with the goal of enhancing therapeutic efficacy and reducing toxicity. The surface expression of the disialoganglioside GD2 is a hallmark of H3K27M gliomas, making it a potential target for CAR T-cell therapy. Although CAR T-cell therapy has demonstrated favorable safety profiles and early efficacy in other solid tumors, there are severe potential adverse consequences associated with immunotherapy for the treatment of tumors within the brain, including obstructive hydrocephalus, elevated intracranial pressure, and neurologic dysfunction. Nevertheless, in a recent phase 1 clinical trial aimed at establishing safety protocols, patients with DMG received GD2 CAR T-cells intravenously followed by intraventricular GD2 CAR T-cells [45]. Three out of four patients were found to exhibit transient clinical and radiographic improvement along with increased proinflammatory cytokine levels in plasma and CSF. These early data suggest that GD2 targeted CAR-T therapy may be a promising therapeutic approach for DIPG, albeit requiring attentive monitoring and proper intervention in case of local or regional inflammation within the brain (Figure 2).

In DIPG, as in other cancers, inhibitory checkpoints restrict the activity of immune cells, including CAR-T-cells, within the tumor microenvironment and pose a challenge that needs to be overcome for effective immunotherapies [76]. Targeting immune checkpoints with inhibitors has been shown to be effective and has contributed to increasing the median average survival in several cancers, particularly in adults. A single-institution, retrospective study assessed the efficacy of PD-1 inhibition (PD-1i) in combination with reirradiation compared to patients who received reirradiation alone, and a slight improvement in median OS was seen (from 22.9 months [reirradiation plus PD-1i] vs. 20.4 months [reirradiation]) [76]. Additionally, an ongoing study is analyzing the effects on DMG tumor progression by introducing the HDAC inhibitor panobinostat with a non-invasive focused ultrasound (FUS) to enhance drug penetration across the BBB [77]. FUS allows a greater amount of drug to enter in close proximity to the tumor, where increased access of the drug to the tumor may provide an enhanced antitumor response.

Tumor vaccines function to trigger an immune response against tumor antigens, such as the mutant H3K27M in DIPG, with promising clinical results. In a recent clinical trial, pediatric patients with DIPG were administered a H3K27M vaccine, followed by immunomonitoring and imaging every 3 months [78]. Results demonstrated that the therapy was well tolerated, and the median OS was 16.1 months for patients who had expansion of H3.3K27M-reactive CD8+ T-cells compared to 9.8 months for those who did not (*p* = 0.05). Immunotherapies for DIPG/DMG show preclinical and early clinical promise, with their ability to harness the immune system and create an anti-tumor immune response, with potential to improve survival outcomes and minimize toxicity.

## 9. Focused Ultrasound

Focused ultrasound (FUS) has emerged as an innovative technique to address the issue of drug delivery and effectiveness in the CNS. With the recent advancements in molecular therapeutic targets and pharmacological therapies for DIPG, the BBB remains a significant obstacle to safe and effective delivery of these agents to the target of interest in the CNS. The BBB, made up of tight junctions of endothelial cells in cerebral microvasculature, is impermeable to a majority of therapeutic agents being trialed for DIPG. The use of MRI-guided FUS for the treatment of CNS tumors has been around for decades, initially introduced with the purpose of ablative therapy with high frequencies [79]. It was not until more recently that FUS has been adapted to be used at lower frequencies with the intention of temporarily disrupting the BBB to allow for drug delivery to the area of interest. Low frequency waves create oscillations that temporarily disrupt the endothelial tight junction, thus increasing permeability of the BBB for the timeframe of drug delivery [80]. Now called low-intensity focused ultrasound (LIFU), the effectiveness of LIFU to increase bioavailability of chemotherapy agents has been shown in numerous preclinical publications, though there remains limited data to date on the effectiveness in human patients [81,82,83,84,85]. In addition to increasing the bioavailability of therapeutic agents via BBB disruption, LIFU can also increase the effectiveness of pharmacological agents by increasing the cytotoxic effects on tumor cells through drug activation, altering the tumor microenvironment and cellular resistance pathways [86].

Strong preclinical data has led to multiple planned and ongoing clinical trials investigating the utility of LIFU for the treatment of DIPG. Syed et al. recently published plans for the first human, early phase 1 and 2 trial studying the use of LIFU for sonodynamic therapy (SDT) with aminolevulinic acid (ALA) for patients with DIPG [87]. The group at Children’s National Hospital is currently conducting the trial with a goal of roughly 18 patients older than 5 years of age with newly diagnosed DIPG who will be divided into dose-escalated cohorts of treatment with ALA and sensitization with SDT. The objective of the study is to investigate the safety of ALA SDT in humans and determine the maximum tolerable dose that can be used for future larger clinical trials. The first patient of the trial has been recruited and has undergone the first phase of treatment. The patient underwent biopsy of the diffuse pontine lesion, confirming the DIPG diagnosis, received standard radiation treatment, and then underwent the first dose of ALA with transfusion 6 h before receiving SDT. SDT was performed under MRI guidance with FUS delivering 220 kHz to 50% of the tumor volume. A one-time sonication was delivered at 50 W with a total of 200 J of energy per focus. A total of 28 sonications were performed to cover half of the tumor volume with plans to return 30 days later to undergo treatment of the other half of the tumor. The patient tolerated the treatment well without any adverse effects. The future results of this study will help guide the protocol for ongoing investigation into the effectiveness of FUS.

Similarly, there is a planned clinical trial (NCT05630209) to be conducted by the Hospital for Sick Children in Toronto in conjunction with Children’s National Hospital that will be investigating the use of MR-guided LIFU to increase the bioavailability and enhance the efficacy of doxorubicin in the treatment of DIPG. This early phase trial is a single-arm, non-randomized trial that will assess the safety and feasibility of this regimen and technique. The study plans to enroll 18 patients between the different sites with the primary endpoint of determining safety by documenting adverse events for the patients utilizing the Common Terminology Criteria for Adverse Events (CTCAE). An additional secondary endpoint of this study will be to determine the effectiveness of the FUS technique by evaluating new contrast enhancement on post-sonication MRI as evidence of disruption of the BBB and presumed bioavailability of the drug. Preliminary data will also be collected regarding changes to tumor size and the PFS of the patients, though these data will primarily help guide future studies and are not an endpoint of the study. Overall, FUS is still in its early development with regards to human use, but initial results are promising.

## 10. Conclusions

DIPG remains a diagnosis with an extremely poor prognosis. While median survival for the disease remains at just about 12 months after diagnosis, there have been promising advances in multiple aspects of DIPG management including diagnosis and treatment. Diagnostic tools are becoming safer and more widely acceptable due to advanced surgical biopsy techniques using stereotactics, robotics, and intraoperative monitoring as well as more accurate liquid biopsy techniques utilizing both CSF and serum samples. Treatment options continue to expand with ongoing trials including radiation, immunotherapy, chemotherapy, and epigenetic modulating therapies that are early in development but have shown promise with the potential of extending survival in these patients. In addition to direct therapies, LIFU in conjunction with developing chemotherapies, has added an additional component to the treatment toolbox to combat DIPG to make these therapies more effective. While there remains an immense amount of work to be accomplished with regards to treatments for DIPG, with more resources dedicated towards advancement of the management of DIPG, there is strong reason for optimism in future developments of impactful treatments for children with DIPG.

## Figures and Tables

**Table 1:** Summary of recent, ongoing, and planned clinical trials for treatment of DIPG.

	Title	Clinical Trial ID	Principal Investigator	Study Design	Agents Investigated	Sample Size	Age Requirement	Summary of Outcomes
Chemotherapy	Combination Therapy for the Treatment of Diffuse Midline Gliomas	NCT05009992	Sabine Mueller, MD, PhD	Phase II	ONC201 (Doradaviprone) and paxalisib	360 patients	2–39 years old	Progression-free survival, overall survival, safety and toxicity, results TBD
	ONC206 for Treatment of Newly Diagnosed, Recurrent Diffuse Midline Gliomas, and Other Recurrent Malignant CNS Tumors (PNOC023)	NCT04732065	Sabine Mueller, MD, PhD	Phase I	ONC206 (analog of ONC201) alone or in combination with radiation therapy	Currently enrolling	2–21 years old	Safety and toxicity, results TBD
Targeted Therapy	Biological Medicine for Diffuse Intrinsic Pontine Glioma (DIPG) Eradication (BIOMEDE)	NCT02233049	Gustave Roussy, MD	Phase II	Post radiation patients treated with erlotinib, everolimus, or dasatinib based on having biomarker EGFR, biomarker mTOR, or no specific biomarker, respectively	233 patients	6 months–25 years old	Median overall survival of 9.0 (7.4–14.3) months, 11.3 (10.3–13.4) months, and 9.4 (7.7–10.7) months for eroltinib, everolimus, and dasatinib, respectively (*p* = 0.45)
	Molecular Profiling for Individualized Treatment Plan for DIPG	NCT02274987	Sabine Mueller, MD, PhD	Phase I/Pilot feasibility	Personilized treatment recommendations based on UCSF 500 gene panel and RNA seq analysis of the tumor	19 patients	3–25 years old	Median overall survival 13. (11.2–18.4) 1 months
	Clinical Benefit of Using Molecular Profiling to Determine an Individualized Treatment Plan for Patients with High-Grade Glioma (PNOC008)	NCT03739372	Sabine Mueller, MD, PhD	Phase I/Pilot feasibility	Non-DIPG DMG patients received personalized treatment following real-time molecular profiling	22 patients	<25 years old	Median overall survival 21.5 (16.8–31.6) months
	A Study of the Drug Selinexor with Radiation Therapy in Patients with Newly-Diagnosed Diffuse Intrinsic Pontine (DIPG) Glioma and High-Grade Glioma (HGG)	NCT05099003	Adam Green, MD	Phase I/II	Selinexor with radiation therapy	210 patient	1–21 years old	To be determined
Radiation Therapy	Stereotactic Biopsy Split-Course Radiation Therapy in Diffuse Midline Glioma, SPORT-DMG Study	NCT05077735	Anita Mahajan, MD	Phase II	Hypo-fractionated radiation therapy, 25 Gy in 10 fractions	20 patients	>1 year old	To be determined
	Reirradiation of Progressive or Recurrent DIPG	NCT03126266	Lucie Lafay-Cousin, MD	Phase II	Non-randomized comparison of conventional fractionation reirradiation with a dose of 30.6 Gy or 36 Gy in recurrent or progressive DIPG	27 patients	Any	To be determined
Epigenetic Modifying Therapy	Vorinostat, Temozolomide, or Bevacizumab in Combination with Radiation Therapy Followed by Bevacizumab and Temozolomide in Young Patients with Newly Diagnosed High-Grade Glioma	NCT01236560	Maryam Fouladi, MD	Phase II/III	Vorinostat, bevacizumab, or temozolomide monotherapy with radiation therapy, followed by temozolomide and bevacizumab combination therapy	90 patients	3–22 years old	1-year EFS for concurrent bevacizumab, vorinostat, or temozolomide with RT of 43.8% (±8.8%), 41.4% (±9.2%), and 59.3% (±9.5%), respectively. No statistically significant difference
Immunotherapy	GD2 CAR T-Cells in Diffuse Intrinsic Pontine Gliomas (DIPG) and Spinal Diffuse Midline Glioma (DMG)	NCT04196413	Michelle Monje, MD, PhD	Phase I	GD2 CAR T-cells intravenously followed by intraventricular GD2 CAR T-cells	4 patients	5–25 years old	3 out of 4 patients exhibited transient clinical and radiographic improvement and increased proinflammatory cytokine levels in plasma and cerebrospinal fluid
	H3.3K27M Peptide Vaccine with Nivolumab for Children with Newly Diagnosed DIPG and Other Gliomas	NCT02960230	Sabine Mueller, MD, PhD.	Phase II	H3K27M vaccine, followed by immunomonitoring and imaging every 3 months	29 patients	3–21 years old	Median overall survival of 16.1 months for patients who had expansion of H3.3K27M-reactive CD8+ T-cells compared to 9.8 months for those who did not (*p* = 0.05)
Focused Ultrasound	Non-Invasive Focused Ultrasound (FUS) with Oral Panobinostat in Children with Progressive Diffuse Midline Glioma (DMG)	NCT04804709	Cheng-Chia Wu, MD, PhD	Phase I	Focused ultrasound with penobinostat	TBD	4–21 years old	To be determined
	Blood–Brain Barrier (BBB) Disruption Using Exablate Focused Ultrasound With Doxorubicin for Treatment of Pediatric DIPG	NCT05630209	Children’s National Group	Phase I	Low-frequency MR-guided focused ultrasound with doxorubicin	18 patients	>5 years old	To be determined
	A Phase 2 Study of Sonodynamic Therapy Using SONALA-001 and Exablate 4000 Type 2.0 in Patients with DIPG	NCT05123534	Children’s National Group	Phase I	Low-frequency MR-guided focused ultrasound with aminolevulinic	18 patients	>5 years old	To be determined
